# Self-initiated lifestyle changes during a fasting-mimicking diet programme in patients with type 2 diabetes: a mixed-methods study

**DOI:** 10.1186/s12875-024-02405-5

**Published:** 2024-05-02

**Authors:** Elske L. van den Burg, Marjolein P. Schoonakker, Bregje Korpershoek, Lara E. Sommeling, Carlijn A. Sturm, Hildo J. Lamb, Hanno Pijl, Mattijs E. Numans, Marieke A. Adriaanse, Petra G. van Peet

**Affiliations:** 1 Department of Public Health and Primary Care, Leiden University Medical Centre (LUMC), Postzone V0-P, Postbus 9600, 2300 RC Leiden, The Netherlands; 2 Department of Radiology, Leiden University Medical Centre (LUMC), Leiden, The Netherlands; 3 Department of Internal Medicine, Leiden University Medical Centre (LUMC), Leiden, The Netherlands; 4 Department of Health, Medical and Neuropsychology, Leiden University, Leiden, The Netherlands

**Keywords:** Type 2 diabetes, Fasting-mimicking diet, Intermittent energy restriction, Periodic fasting, Diet, Dietary behaviour, Physical activity, Mixed-methods, Self-initiated behavioural change

## Abstract

**Background:**

Lifestyle changes, especially regarding diet quality and physical activity, are important in the management of type 2 diabetes (T2D). This mixed-methods study explores self-initiated lifestyle changes in patients with T2D who followed a periodic fasting-mimicking diet (FMD).

**Methods:**

Quantitative data were obtained from the Fasting In diabetes Treatment trial (November 2018 to August 2021) in which 100 participants with T2D, using metformin only or no medication, were randomised to receive a monthly 5-day FMD for twelve months next to usual care, or usual care only. Diet quality and physical activity questionnaires were completed at baseline, six and twelve months. Changes over time were analysed using linear mixed models. Focus groups were organized with FMD participants to explore experiences regarding self-initiated lifestyle changes. The qualitative data was analysed using the Theoretical Domains Framework.

**Results:**

Questionnaires were available from 49 FMD participants and 43 controls. No differences in diet quality were found. Total physical activity in the FMD participants changed from 34.6 to 38.5 h per week (h/wk) from baseline to twelve months, while in controls it changed from 34.9 to 29.0 h/wk (between group difference, p = 0.03). In six focus groups with FMD participants (n = 20), individual participants perceived the FMD as an encouragement for (minor) lifestyle changes. There were no barriers to behaviour change related to the FMD. Important facilitators of healthy behaviour were an increase in awareness of the impact of lifestyle on health (*knowledge*), better physical fitness (*physical*) and health improvement (*reinforcement*). Facilitators unrelated to the FMD included family support (*social influences*) and opportunities in the neighbourhood (*environmental context and resources*), while barriers unrelated to the FMD were experiencing health problems (*physical*) and social events (*social influences*).

**Conclusions:**

Using an FMD for five consecutive days per month did not affect diet quality in between FMD periods in quantitative analysis, but increased the number of hours per week spent on physical activity. Qualitative analysis revealed self-initiated improvements in both diet quality and physical activity in individual participants using an FMD. Healthcare professionals could use an FMD programme as a ‘teachable moment’ to stimulate additional lifestyle changes.

**Trial registration:**

ClinicalTrials.gov; NCT03811587. Registered 22 January 2019.

**Supplementary Information:**

The online version contains supplementary material available at 10.1186/s12875-024-02405-5.

## Background

Type 2 diabetes (T2D) is a major societal challenge as its prevalence and burden of disease continues to rise worldwide [1]. Lifestyle factors play an important role in the development of T2D [2]. Various dietary programs have shown to improve glycaemic control [3–5]. Increased physical activity can also optimize glycaemic control, even when not accompanied with weight loss [6, 7]. Therefore, interventions that promote and support healthy dietary changes and regular physical activity are fundamental to the management of T2D.

Despite the known beneficial effects, it is difficult to maintain healthy lifestyle changes [8–10]. Consequently, new lifestyle interventions are being developed that may be easier to maintain. Examples are intermittent energy restriction (IER) and periodic fasting, as alternatives to continuous energy restriction [11]. Periods of significant energy restriction alternated with periods of unrestricted eating can result in improved glycaemic control and weight loss in patients with T2D [12, 13]. A specific form of periodic fasting is a fasting-mimicking diet (FMD). Recently, positive effects of the periodic use of an FMD have been found on weight, fasting plasma glucose and glycated haemoglobin (HbA1c) in patients with T2D [14, 15]. An FMD mimics the effects of water-only fasting while minimizing its burden by allowing light meals to be consumed during the fasting days [16]. Furthermore, the fasting periods usually last no more than four to seven days and the frequency of the fasting period is no more than once per month. FMDs are low-energy, plant-based, formula diets which are low in sugar and protein and primarily comprise complex carbohydrates and healthy fats [17]. The macronutrient composition is combination with the low-energy content cause the fasting-mimicking effects of the diet, which include for example a reduction of serum glucose [18].

In the Fasting In diabetes Treatment (FIT) trial, a monthly 5-consecutive day FMD programme was integrated in regular primary care for patients with T2D who use metformin as the only glucose-lowering drug and/or diet for glycaemic control [19]. When comparing the FMD group to the control group after 12 months, the FMD group used significantly less glucose-lowering medication and mean HbA1c levels, body weight, body fat percentage and waist circumference were lower, while fat-free mass did not change [20].

Knowledge about the influences of the periodic use of an FMD on additional self-initiated lifestyle changes is lacking. Some studies investigating short-term effects of IER suggest that it may be accompanied by self-initiated changes of dietary intake and physical activity [21–26]. Since an FMD only takes four to seven consecutive days a month, the question arises how following an FMD programme influences lifestyle in between FMD cycles. We hypothesize that following an FMD programme might influence lifestyle in patients with T2D, especially with regard to diet quality and physical activity. We aim to investigate this in the FIT trial using a mixed methods design, with quantitative data of validated questionnaires on changes in diet quality and physical activity and qualitative data on participants’ experiences from focus group discussions.

## Methods

### Trial design

The present mixed-methods study has a convergent parallel design, in which the quantitative and qualitative data were collected simultaneously and analysed separately [27]. The aim of this design was to create two mutually exclusive sets of data that inform each other. We assessed additional self-initiated changes in diet quality and physical activity with quantitative analysis of validated questionnaires. Furthermore, we assessed participants’ experiences with self-initiated lifestyle changes while following an FMD using qualitative analysis of focus group discussions. We identified barriers and facilitators involved in this process, and explored participant wishes regarding additional support from healthcare professionals.

The present study was conducted in the context of the FIT trial, which is a randomised, controlled, assessor-blinded intervention trial conducted between November 2018 and August 2021 at the Leiden University Medical Centre (LUMC) in the Netherlands [19]. Participants were eligible when they were diagnosed with T2D, had a BMI ≥ 27 kg/m^2^ and were aged > 18 years and < 75 years. They were included if they were treated in primary care with lifestyle advice alone (HbA1c above 6.5%; 48 mmol/mol), or treated with lifestyle advice plus metformin as the one and only glucose-lowering drug (regardless of their HbA1c). After randomisation, participants in the FMD group received twelve 5-consecutive day FMD cycles on a monthly basis for one year as an adjunct to usual care. The control group received usual care only. The FMD consisted of complete meal replacement products, mainly soups, bars and tea (Appendix 1). Caloric content and macronutrient composition were as follows; day 1 contained ~ 1100 kcal (10% protein, 56% fat and 34% complex carbohydrate); days 2–5 were identical and provided ~ 750 kcal (9% protein, 44% fat, 47% complex carbohydrate). Participants in the FIT trial were not given any information or instruction to impose lifestyle changes other than adherence to the monthly 5-day FMD. Details of the study design and exclusion criteria can be found in the study protocol [19]. Enrolment, allocation, follow-up and effects on metabolism and anthropometrics are described in detail elsewhere [20]. The protocol and amendments were approved by the Medical Research Ethics Committee of the LUMC. The trial was conducted according to the principles of the Declaration of Helsinki, the Medical Research Involving Human Subjects Act, and the standards of Good Clinical Practice. The CONSORT 2010 guidelines (Appendix 2) [28] as well as the consolidated criteria for the reporting of qualitative research [29] were used to guide the conduct and reporting of this study. All participants provided written informed consent. The trial was prospectively registered on the 22nd of January 2019 in ClinicalTrials.gov, NCT03811587.

### Quantitative study

#### Diet quality

Diet quality was assessed at baseline, six and twelve months by using the web-based Eetscore Food Frequency Questionnaire (Eetscore FFQ), which is a 40-item validated screening food frequency questionnaire using the Dutch Healthy Diet 2015-index (DHD2015-index) [30, 31]. The DHD2015-index assesses diet quality and adherence to the Dutch dietary guidelines of 2015 [32]. The Eetscore FFQ consists of 16 components, examples are ‘vegetables’, ‘red meat’, ‘fats and oils’ and ‘alcohol’. For each component a score is calculated ranging between 0 and 10, resulting in a total adherence score to the Dutch dietary guidelines of 0 (minimal adherence) to 160 (maximal adherence). A higher (sub) score indicates a better diet quality. At each time point, the reference period for the participants was the previous month, excluding the FMD period. The main outcome was the change in total score of the Eetscore FFQ over time. Furthermore, the changes in each individual food component over time were analysed.

#### Physical activity

Physical activity was measured at baseline, six and twelve months using the Short QUestionnaire to ASsess Health-enhancing physical activity (SQUASH), a 15-item validated questionnaire to assess the level of habitual physical activity during an average week in the Dutch population [33, 34]. The questions are structured in frequency (days per week), duration (average time per day) and intensity (light, moderate or vigorous) across different domains (commuting, work and school, household, leisure time and sports). The intensity of activities was based on the Metabolic Equivalent of a Task (MET) derived from the Compendium of Physical Activities of 2011 [35]. The following results were calculated: hours per week (h/wk) spent on physical activity of low (MET < 3.0), moderate (MET 3.0–5.9) and vigorous intensity (MET ≥ 6.0), and total hours per week spent on physical activity [35]. Low-intensity physical activity included home activities such as mopping or washing dishes; moderate-intensity physical activity included activities such as walking and gardening activities; and high-intensity physical activity included activities such as cycling and running [35]. The outcomes are changes in hours per week spent on total, low-intensity, moderate-intensity, and high-intensity physical activity over time.

#### Statistical analysis

Outcomes were summarized using mean and standard deviation (SD) or median and interquartile range (IQR) in case of an asymmetric distribution. Changes over time of the outcomes from the Eetscore FFQ and SQUASH were estimated with linear mixed models at six months and twelve months, relative to baseline (intention-to-treat analysis). The outcome model included fixed effects for time-by-arm interaction terms with random effects for individual participants. Statistical analyses were performed using Rstudio version 4.3.1 for Windows. Figures were created in GraphPad Prism version 9.3.1 for Windows.

### Qualitative study

#### Focus groups

We organized focus group discussions with the participants of the FMD group to explore their experiences. Focus groups were chosen as a research method, as it allows participants to discuss their experiences, perceptions and strategies for lifestyle changes. During these focus groups (90–120 min), two main topics were discussed with a ten-minute break in between: 1) adherence to the FMD (these results will be described elsewhere); and 2) additional self-initiated lifestyle changes while following an FMD (current study). To complement information from questionnaires, participants were asked about their experiences with self-initiated lifestyle changes while following an FMD. The aim was to gain insight into individual participant experiences.

#### Participants

Participants eligible for the focus groups were those enrolled in the FMD group who had completed the follow-up period. FMD participants were purposively sampled to ensure diversity in gender, age, BMI and adherence to the FMD. Participants were invited to participate by telephone, after which they received further information by letter. An additional written informed consent specifically for the focus group discussions was provided by the participants prior to the focus groups, and verbal informed consent was recorded at the beginning of each focus group.

#### Data collection

Due to the coronavirus disease 2019 (COVID-19) outbreak, the focus groups were conducted online through the video platform “Jitsi” (https://meet.jit.si/). As focus groups had to be conducted online, we limited each group to a maximum of four participants, which is a smaller number of participants than is used in traditional face-to-face settings [36]. The reason was that we expected that online interaction would be more difficult compared to face-to-face interactions and that smaller groups would facilitate active online participation. All focus groups were organized between December 2020 and April 2021, and were conducted by a senior investigator who was an experienced moderator (PP) and were assisted by two other researchers (EB or MS, and CS) observing the interviews and making field notes. After each focus group, the researchers shared notes and discussed the main themes of the focus group in order to determine whether or not data saturation was reached.

The content of the focus groups was guided by a semi-structured questionnaire developed by the research team to facilitate the discussion (Appendix 3). The current study analyses the following questions relating to lifestyle changes: 1) What were participant experiences concerning additional self-initiated lifestyle changes while following an FMD?; 2) Which barriers and facilitators were involved?; 3) Did participants wish for additional support from healthcare professionals to stimulate additional lifestyle changes?

#### Analysis

To evaluate the representativeness of our focus group participants for the whole group of FMD users, we compared their baseline data and number of completed FMD cycles with those of the other FMD group participants. Continuous variables were compared by using the independent t-test, or the Mann–Whitney U test if the assumption of normality was violated. Categorical outcomes were analysed with the chi-square test, or the Fisher’s exact test if the assumptions of the chi-square test were violated.

All focus groups were audio-recorded and manually transcribed verbatim (CS). Names and other identifying data were omitted from the transcript and replaced with the study ID. Two researchers (EB, MS) reviewed all transcripts to correct any discrepancies between the audio recording and the transcript. The software program Atlas.ti version 22 for Windows was used for analysis.

Two researchers (EB, LS) independently open coded the text by assigning strings or words/phrases (codes) to segments of text (quotes). Quotations and final codes were discussed (EB, MS, LS, PP), and a code list was established. The code list including the quotations was thereafter discussed within the research team. Codes regarding barriers and facilitators to lifestyle changes were mapped onto the Theoretical Domains Framework (TDF), which is a specification of the Capability, Opportunity and Motivation Behaviour (COM-B) model [37–39]. While the TDF was originally developed to analyse the behaviour of healthcare professionals [38], it is currently also used to analyse changing patient behaviour, including lifestyle changes [40–42].

## Results

### Quantitative study

#### Participant characteristics

**Fig. 1:**
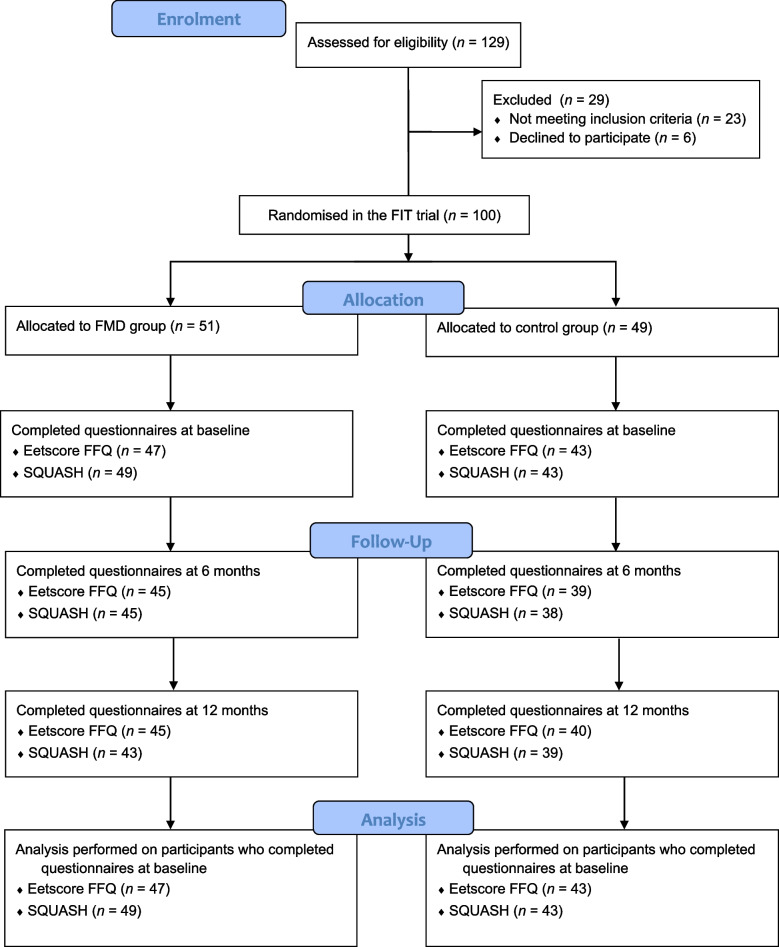
Flow chart of participant inclusion in the FIT trial, followed by number of participants who completed the Eetscore FFQ and the SQUASH at baseline, six months and twelve months

For the quantitative study, data were available of 92 participants who completed the SQUASH at baseline (FMD group *n* = 49, control group *n* = 43) and 90 participants who completed the Eetscore FFQ at baseline (FMD group *n* = 47, control group *n* = 43, Fig. 1). In general, the FMD group (*n* = 49) and the control group (*n* = 43) were similar in terms of baseline characteristics (Table 1).

**Table 1:** Baseline characteristics

	**FMD group (*n* = 49)**	**Control group (*n* = 43)**
**Demographics**		
Age (years), mean ± SD	63.6 ± 8.1	62.2 ± 8.5
Sex, *n* (%)		
Male	26 (53.1)	22 (51.2)
Female	23 (46.9)	21 (48.8)
Level of education, *n* (%)		
Low	20 (40.8)	15 (34.9)
Intermediate	13 (26.5)	13 (30.2)
High	14 (28.6)	15 (34.9)
Unknown	2 (4.1)	0 (0.0)
Current smoker, *n* (%)	4 (8.2)	4 (9.3)
Alcohol use, *n* (%)	25 (51.0)	22 (51.2)
Vegetarian, *n* (%)	2 (4.1)	0 (0)
**Type 2 diabetes**		
Time since diagnosis T2D (years), median (IQR)	4 (3–12)	6 (3–10)
HbA1c (%), mean ± SD	6.9 ± 0.8	7.1 ± 1.1
HbA1c (mmol/mol), mean ± SD	52.2 ± 9.3	53.7 ± 12.2
Fasting glucose (mmol/L), mean ± SD^a^	8.3 ± 1.9	8.8 ± 1.8
Use of glucose-lowering medication		
Metformin, *n* (%)	46 (93.9)	36 (83.7)
Metformin dose, median (IQR)	1000 (500 – 1700)	750 (500 – 1000)
**Anthropometrics**		
Weight (kg), mean ± SD	100.5 ± 15.3	99.2 ± 14.3
BMI (kg/m^2^), median (IQR)	31.3 (29.2 – 35.7)	31.9 (29.8 – 34.3)
**Questionnaires**		
Eetscore FFQ (range 0–160)		
Total score, mean ± SD^b^	104.5 ± 15.9	98.8 ± 20.9
SQUASH		
Total physical activity (h/wk), mean ± SD	34.6 (16.8)	34.9 (21.0)
Low-intensity physical activity (h/wk), mean ± SD	9.9 (10.0)	10.0 (8.7)
Moderate-intensity physical activity (h/wk), mean ± SD	22.1 (17.4)	21.1 (18.9)
High-intensity physical activity (h/wk), mean ± SD	2.7 (3.3)	3.8 (5.3)

Baseline characteristics of the participants of the FIT trial who completed at least one of the questionnaires (Eetscore FFQ or SQUASH) at baseline (*n* = 92). Data are presented as mean ± SD, median (IQR) or number (*n*) with percentage (%)

*BMI* Body Mass Index, *Eetscore FFQ* Eetscore Food Frequency Questionnaire, *FMD* Fasting-mimicking diet, *HbA1c* Glycated haemoglobin, *h/wk* Hours per week, *IQR* Interquartile range, *n* Number, *SD* Standard deviation, *SQUASH* Short QUestionnaire to ASsess Health-enhancing physical activity, *T2D* Type 2 diabetes

^a^Two participants did not arrive in fasting condition, missing data: FMD group *n* = 1; control group *n* = 1

^b^Missing data: FMD group *n* = 2

#### Diet quality

**Fig. 2:**
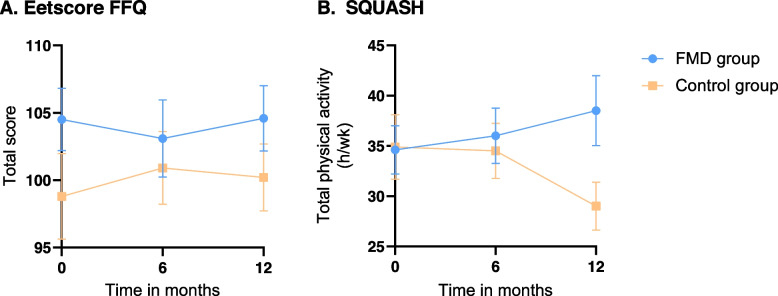
Results of the quantitative analysis of the Eetscore FFQ and the SQUASH over time. Values are presented as mean ± standard error of the mean. **a** The mean total score of the Eetscore FFQ (range 0-160) per group at baseline, six months and twelve months. **b** The mean time spent on total physical activity per group (h/wk) at baseline, six months and twelve months. Eetscore FFQ Eetscore Food Frequency Questionnaire, FMD Fasting-mimicking diet, h/wk Hours per week, SQUASH Short QUestionnaire to ASsess Health-enhancing physical activity

Regarding diet quality, there was no significant effect of following an FMD on the total score of the Eetscore FFQ over time (Table 2, Fig. 2a). Furthermore, there were no significant effects of following an FMD on any of the sub-scores of the Eetscore FFQ over time (Appendix 4).

**Table 2:** Analyses of the Eetscore FFQ and the SQUASH over time using linear mixed models

	**FMD group**		**Control group**		**Estimated effect (95% CI)**	***p*-value**
	**n**	**Mean (SD)**	**n**	**Mean (SD)**		
**Eetscore FFQ (Total score, range 0–160)**						
Baseline	47	104.5 (15.9)	43	98.8 (20.9)		
6 months	45	103.1 (17.9)	39	100.9 (16.9)	-3.0 (-8.1 to 2.1)	0.25
12 months	45	104.6 (15.4)	40	100.2 (15.8)	-1.1 (-6.2 to 4.0)	0.69
**SQUASH**						
Total physical activity (h/wk)						
Baseline	49	34.6 (16.8)	43	34.9 (21.0)		
6 months	45	36.0 (18.5)	38	34.5 (16.9)	0.3 (-7.6 to 8.2)	0.94
12 months	43	38.5 (22.9)	39	29.0 (14.9)	9.1 (1.2 to 17.1)	0.03
*Sub-scores*						
Low-intensity physical activity (h/wk)						
Baseline	49	9.9 (10.0)	43	10.0 (8.7)		
6 months	45	10.9 (8.7)	38	11.4 (7.9)	-1.1 (-4.4 to 2.2)	0.50
12 months	43	11.4 (11.8)	39	8.7 (6.4)	2.3 (-0.9 to 5.6)	0.17
Moderate-intensity physical activity (h/wk)						
Baseline	49	22.1 (17.4)	43	21.1 (18.9)		
6 months	45	21.1 (18.0)	38	18.6 (14.6)	0.8 (-5.8 to 7.4)	0.82
12 months	43	23.7 (19.4)	39	16.8 (11.8)	5.7 (-0.9 to 12.4)	0.10
High-intensity physical activity (h/wk)						
Baseline	49	2.7 (3.3)	43	3.8 (5.3)		
6 months	45	4.0 (4.5)	38	4.5 (5.7)	0.4 (-1.2 to 2.0)	0.63
12 months	43	3.5 (4.0)	39	3.5 (5.2)	0.9 (-0.7 to 2.6)	0.28

Linear mixed models were computed with time, intervention and time-by-intervention interaction as fixed-effects, and individual participants as random effect

*CI* Confidence interval, *Eetscore FFQ* Eetscore Food Frequency Questionnaire, *FMD* Fasting-mimicking diet, *h/wk* Hours per week, *SD* Standard deviation, *SQUASH* Short QUestionnaire to ASsess Health-enhancing physical activity

#### Physical activity

Total physical activity in the FMD group changed from 34.6 h/wk at baseline to 38.5 h/wk at twelve months, while in the control group total physical activity changed from 34.9 h/wk at baseline to 29.0 h/wk at twelve months (between group difference, *p* = 0.03; Table 2, Fig. 2b). All sub-scores of physical activity show a non-significant increase in h/wk in the FMD group compared to the control group (Table 2).

### Qualitative study

Six focus groups were conducted with three to four participants per focus group. Data saturation was reached in the sixth focus group. The focus group participants (*n* = 20) were similar compared to the other FMD participants (*n* = 29) regarding baseline characteristics, except for the number of completed FMD cycles, as in the focus groups a relatively high number of FMD participants completed all FMD cycles (median of twelve cycles completed in the focus group participants vs median of eleven cycles completed in the other FMD participants; *p* = 0.02; Appendix 5).

Data from the focus groups are presented according to the three research questions: 1) What were participant experiences concerning additional self-initiated lifestyle changes while following an FMD?; 2) Which barriers and facilitators were involved?; 3) Did participants wish for support from healthcare professionals to stimulate additional lifestyle changes? The data from the second research question is mapped onto the combined COM-B and TDF model. The corresponding TDF domain is presented in italic between brackets *(TDF domain)*.

#### Participants’ experiences

Many participants perceived the FMD as an encouragement to a spontaneous change of (minor) aspects of their lifestyle, that contributed to a healthier diet or an increase in physical activity. For example, one participant said: *"I have not gained weight yet [since the end of the FIT trial], I am off medication, off the diabetes medication, also half of my blood pressure medication has been stopped. I have learned to eat differently. As far as my rheumatism goes, I'm doing incredibly well. I can move much better and I'm incredibly happy about it. I have much less pain." [FG4, female]* For some others, the FMD was not a trigger to focus on lifestyle changes. Some of these participants indicated that they were already paying attention to a healthy lifestyle, which motivated them to participate in the trial in the first place.

#### Barriers and facilitators

**Fig. 3:**
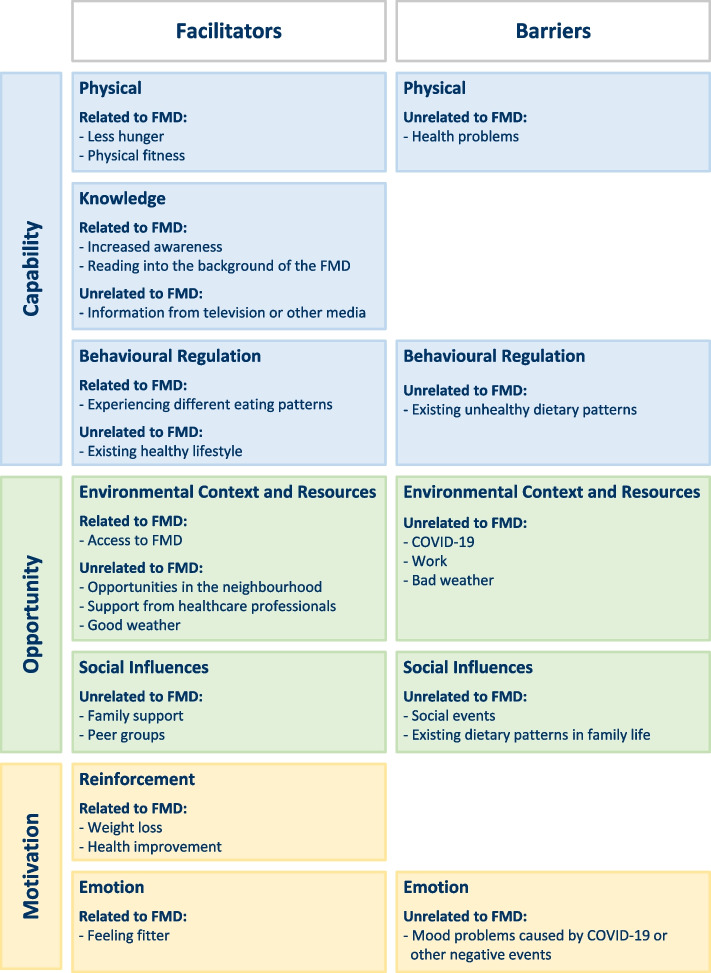
Main barriers and facilitators for spontaneous lifestyle changes while following an FMD that are either related or unrelated to the FMD, as reported by focus group participants. Results are mapped onto the Theoretical Domains Framework (TDF) (in *italics*) combined with the Capability, Opportunity, and Motivational Behaviour (COM-B) model. Since barriers and facilitators overlapped between changes in diet and changes in physical activity, they are grouped in one figure

In the focus group discussions, barriers and facilitators for additional self-initiated lifestyle changes while following a periodic FMD were identified (Fig. 3, Appendix 6). Some facilitators were directly related to following the FMD, while others were unrelated to the FMD. None of the identified barriers were directly related to following the FMD.

Identified barriers and facilitators were generally discussed specifically in the context of one of two types of behaviour: changes in diet and changes in physical activity. Results are therefore presented below for these two behaviours separately.

##### *Changes in diet*

Participants described self-initiated dietary changes in the periods between FMD cycles. The FMD acted as a facilitator in this process. For example, participants experienced less hunger on non-fasting days (*physical*): *"Yes, [I eat] less, anyway, I get satiated much faster. I also really feel that my stomach has become a bit smaller." [FG1, female].* Other facilitating aspects of the FMD were experiencing a new eating pattern (*behavioural regulation*) and increased knowledge about food and diets for example by reading into the background of the FMD (*knowledge*).Awareness was an important facilitator (*knowledge*): participants expressed that following the FMD increased their awareness of the association between lifestyle and health, and the importance of healthy eating for the management of T2D. “*Well, because of the [FMD] boxes that I had, I started to realize that, uh… that what you eat and drink affects your health. […] For the last two months or so, I have been eating less carbohydrates. My diet is not completely carbohydrate free, but I am eating less carbohydrates. And I like it. I am losing weight and I am feeling better.” [FG4, male]*.

A facilitator unrelated to the FMD was social support from family members or friends whereas in contrast, social events were also mentioned as a barrier to maintain healthy dietary changes (*social influences*): *“For me it’s difficult, uh, we have just had the holidays, so then I have gained weight again." [FG4, female].* Another barrier unrelated to the FMD was experiencing negative life events (for example COVID-19 or temporary health problems), which people mentioned as reasons to increase consumption of snacks and comfort food (*emotions*).

##### *Changes in physical activity*

Following an FMD was experienced as a facilitator to increase physical activity as it led to more physical fitness and weight loss (*reinforcement*): *"This diet makes me more able to go for a walk, makes me fitter so that I can walk. […] I was one hundred and twenty kilos, now I think I’m about one hundred and four, […] that brings a lot in terms of fitness, that uh, you can do a lot more, you can walk longer, less injuries, and more stuff like that." [FG1, male]*. An increased awareness of the association between health and physical activity was also mentioned as a result of following an FMD (*knowledge*): *"For me, thinking about physical activity has definitely changed. […] I have become more aware of, for example, taking the stairs. I have bought an activity tracker, that is very stimulating.” [FG1, male]*.

Besides the effects of the FMD, various other factors were perceived to facilitate physical activity. Examples are opportunities in the neighbourhood (nature or a gym) or owning a dog (*environmental context and resources*). Also, aspects like walking with friends, family or peers were mentioned as facilitators for physical activity (*social influences*). On the other hand, social factors were sometimes perceived as a barrier to physical activity, for example when participants experienced peer pressure (*social influences*). COVID-19 was also often mentioned as a barrier for physical activity: participants stayed indoors more often out of fear of becoming ill, and could not visit the gyms (*environmental context and resources*). Work-related barriers were also mentioned, like having a sedentary job (*environmental context and resources*).

#### Support from healthcare professionals

When discussing lifestyle changes in the focus groups, none of the participants spontaneously mentioned the role of healthcare professionals. At the end of the focus groups, participants were explicitly asked if they wished for additional support from healthcare professionals (Appendix 3). Participants often stated that they did not require additional support from healthcare professionals. A few participants did express a need for more consistent support from healthcare professionals in creating healthy dietary habits in addition to following an FMD.

## Discussion

In this exploratory study, we looked at additional self-initiated lifestyle changes in patients with T2D who followed an FMD programme for five consecutive days per month for twelve months. The quantitative analyses did not show an effect of following an FMD on diet quality. We found a positive effect of following an FMD on total hours of physical activity after twelve months.

As far as we know, our trial is the first to examine the relation between following a periodically applied FMD and self-initiated changes in lifestyle. However, there is some quantitative data from other IER diets, with varying results. Two trials offering additional dietary counselling in addition to a fasting window, show a reduction of energy intake on non-restricted days [22, 23]. In other trials, dietary quality in between energy restricted periods did not change at all [24, 43, 44]. Advice regarding physical activity during IER appears to be guiding actual change, as changes were reported when participants were encouraged to increase physical activity [12, 45] while there was no change when they were instructed not to change their physical activity level [23, 46]. Comparison of studies is complicated due to different types of dietary interventions. Also, these studies did not have diet quality and physical activity as primary outcomes, and sometimes lack power to draw conclusions on these outcomes. Another interesting finding in our study, is that total hours of physical activity decline in the control group but increase in the FMD group. During the follow-up period of the FIT trial, the prolonged restrictive measures during the COVID-19 pandemic played a role. Other studies have shown that during the COVID-19 pandemic, there was a decrease in physical activity globally, also in patients with T2D [47–49]. Notably, the FMD group showed an increase in physical activity despite the COVID-19 restrictions, suggesting that following an FMD programme might have had a protective effect regarding physical inactivity.

The focus group discussions revealed (minor) changes in lifestyle contributing to a healthier diet or an increase in physical activity in some participants. None of the identified barriers to lifestyle change was directly related to following the FMD. Rather, key barriers involved experiencing health problems (*physical*) and social events (*social influences*). Although increased awareness of the importance of lifestyle for health (*knowledge*) was often mentioned as one of the facilitators of changes in diet or physical activity, it was not always accompanied by an actual change in lifestyle. Other important facilitators of healthy behaviour related to the FMD were better physical fitness (*physical*) and health improvement (*reinforcement*). Facilitators unrelated to the FMD included family support (*social influences*) and opportunities in the neighbourhood (*environmental context and resources*).

We have found no studies of qualitative research describing the impact of periodic use of an FMD on changes in lifestyle, though previous studies examining facilitators and barriers of lifestyle change may provide clues to explain our findings. Skoglund et al. [50] reported that increasing awareness of the importance of lifestyle for health, disease and its related risks can positively affect the motivation for lifestyle changes, sometimes, but not always, leading to small changes in lifestyle. Accordingly, participants in our study often indicated increased awareness of health risks as a driver of lifestyle change. Regarding the barriers and facilitators unrelated to the FMD, the factors found in this study were actually very comparable to barriers and facilitators identified by patients and healthcare professionals in other studies involving lifestyle changes: family support (*social influences*), difficulty in changing well-established habits (*behavioural regulation*), physical health and fitness (*physical*), and weather and work-related issues (*environmental context and resources*) [51–53].

### Strengths, limitations and future research

Strengths of our research include the combination of quantitative and qualitative methods, where the quantitative analysis yields actual lifestyle changes, while the qualitative analysis gives a more in-depth insight into participant experiences and barriers and facilitators involved in the process of lifestyle changes. Regarding the focus groups, investigator triangulation in the data collection and analysis, and the use of direct quotations, enhanced the reliability of our results.

However, there were also some limitations. Since additional self-initiated lifestyle changes were not the primary aim of the FIT trial, it was not designed or powered to detect changes in outcomes of the Eetscore FFQ or the SQUASH. Results should therefore be considered exploratory. Moreover, participants who were included in the FIT trial were interested in following the FMD programme, which could indicate more interest in lifestyle changes than the general population. Furthermore, the Eetscore FFQ is designed to measure diet quality based on Dutch dietary guidelines of 2015, which is not specific for patients with T2D [31, 32]. This might explain the absence of a change in diet quality in the present study. Also, both the Eetscore FFQ and the SQUASH rely on self-reporting, which influences their reliability. Particularly concerning physical activity, we found high baseline levels which not only led to little room for improvement but might also indicate that participants were inclined to provide socially desirable responses. Additionally, these questionnaires inquired about activities within the month preceding the study visit, thereby potentially introducing recall bias. A potential limitation of the focus groups is that the participants differed from the rest of the FMD group with respect to the number of completed boxes. The focus group participants completed more boxes, which means that their compliance was overall better than the compliance of the other FMD participants. Furthermore, recall bias may have played a role in the focus groups, since all participants had already finished the follow-up period and were not actually following the FMD at the moment.

For future research, we suggest to power IER and FMD studies on changes in diet quality and physical activity questionnaires, and to develop food frequency questionnaires more sensitive for dietary changes specific for patients with T2D. Furthermore, the use of activity trackers can be considered in trials concerning FMDs in order to obtain more objective data on physical activity. In addition, future research could combine following an FMD with an additional behavioural change intervention taking the barriers and facilitator found in this study into account.

### Clinical implications

For healthcare professionals in primary care, it is important to know that following an FMD leads to more awareness about the implications of lifestyle for health in patients with T2D (*knowledge*), but that awareness does not automatically lead to actual changes in lifestyle in all patients. However, following such programme could potentially be used by healthcare professionals as a ‘teachable moment’ to stimulate additional lifestyle changes [54, 55], since patients following an FMD may be more receptive towards lifestyle advices and more motivated to change their lifestyle. The positive effect of following an FMD on total hours per week spent on physical activity can be beneficial for patients with T2D, since any increase in physical activity is associated with improved health outcomes [56, 57]. When patients are following an FMD, healthcare professionals could make use of the identified facilitators, by paying attention to the reinforcing value of experiencing better physical fitness (*physical*) and health improvements (*reinforcement*). Furthermore, they could involve family in lifestyle treatment (*social influences*) and discuss opportunities in the neighbourhood that stimulate healthy behaviour (*environmental context and resources*). Regarding key barriers, healthcare professionals could optimize treatment of other health problems (*physical*) and discuss social events and how to deal with them (*social influences*).

## Conclusions

Quantitative analyses did not show an effect on diet quality in between FMD periods, but there was a positive effect of following a periodically applied FMD for five consecutive days per month on total hours per week spent on physical activity. In qualitative analysis of focus groups we found that individual participants reported self-initiated improvements in both diet quality and physical activity while following the periodic FMD. The results of this study show that following an FMD increases awareness of the impact of lifestyle on health, but that awareness does not automatically lead to self-initiated lifestyle changes. Healthcare professionals could use an FMD programme as a ‘teachable moment’ to stimulate additional lifestyle changes, since FMD participants may be more receptive towards lifestyle advices and more motivated to change their lifestyle. Healthcare professionals could use the identified barriers and facilitators in this study, for example by paying attention to better physical fitness experienced by FMD participants, by involving family members, and by optimizing treatment of other health problems.

### Supplementary Information

**Additional file 1:** **Appendix 1.** Example meal plan. **Appendix 2.** CONSORT 2010 checklist. **Appendix 3.** Semi-structured questionnaire for focus group discussions in the FIT trial. **Appendix 4.** Analyses of the sub-scores of the Eetscore FFQ over time. **Appendix 5.** Characteristics of focus group participants compared to the other FMD participants. **Appendix 6.** Results from focus group discussions.

## Data Availability

The datasets used during the current study are available upon reasonable request. Requests for access to data should be sent to the FIT trial corresponding email (fit@lumc.nl). All proposals requesting data access will need to specify how the data will be used, and all proposals will need approval of the trial co-investigator team before data release.

## Abbreviations

BMI: Body Mass Index
CI: Confidence interval
COM-B: Capability, Opportunity and Motivation Behaviour model
COVID-19: Coronavirus disease 2019
Eetscore FFQ: Eetscore Food Frequency Questionnaire
FIT trial: Fasting In diabetes Treatment trial
FMD: Fasting-mimicking diet
h/wk: Hours per week
HbA1c: Glycated haemoglobin
IER: Intermittent energy restriction
IQR: Interquartile range
LUMC: Leiden University Medical Centre
MET: Metabolic Equivalent of a Task
N: Number
SD: Standard deviation
SQUASH: SHort QUestionnaire to Asses Health-enhancing physical activity
T2D: Type 2 diabetes
TDF: Theoretical Domains Framework
