# Supplementary material for: Self-initiated lifestyle changes during a fasting-mimicking diet programme in patients with type 2 diabetes: a mixed-methods study
Source: BMC Prim Care. 2024 May 2;25:148. doi: 10.1186/s12875-024-02405-5 (PMC11067095; doi:10.1186/s12875-024-02405-5)
Supplement: Supplementary file 1 — Additional file 1: Appendix 1. Example meal plan. Appendix 2. CONSORT 2010 checklist. Appendix 3. Semi-structured questionnaire for focus group discussions in the FIT trial. Appendix 4. Analyses of the sub-scores of the Eetscore FFQ over time. Appendix 5. Characteristics of focus group participants compared to the other FMD participants. Appendix 6. Results from focus group discussions. [file 12875_2024_2405_MOESM1_ESM.pdf]

Supplementary Material

Self-initiated lifestyle changes during a fasting-mimicking diet programme in patients with type 2 diabetes: a mixed-methods study

E.L. van den Burg[1], M.P. Schoonakker[1], B. Korpershoek[1], L.E. Sommeling[1], C.A. Sturm[1], H.J. Lamb[2], H. Pijl[1,3], M.E. Numans[1], M.A. Adriaanse[1,4], P.G. van Peet[1]

Table of contents

Appendix 1 2

Example meal plan 2

Appendix 2 3

CONSORT 2010 checklist 3

Appendix 3 7

Semi-structured questionnaire for focus group discussions in the FIT trial 7

Appendix 4 9

Analyses of the sub-scores of the Eetscore FFQ over time 9

Appendix 5 11

Characteristics of focus group participants compared to the other FMD participants 11

Appendix 6 12

Results from focus group discussions 12

Appendix 1

Example meal plan

	Day 1	Day 2	Day 3	Day 4	Day 5
**Breakfast**	Tea<br>Nut bar<br>Algal Oil capsule	Tea<br>Nut bar	Tea<br>Nut bar	Tea<br>Nut bar	Tea<br>Nut bar<br>Algal Oil capsule
**Lunch**	Tomato Soup<br>Olives<br>Kale crackers<br>Vitamin capsule	Tea<br>Mushroom Soup<br>Olives<br>Vitamin capsule	Tea<br>Tomato Soup<br>Kale Crackers<br>Vitamin capsule	Tea<br>Vegetable Soup<br>Olives<br>Vitamin capsule	Tea<br>Tomato Soup<br>Kale Crackers<br>Vitamin capsule
**Afternoon**	Tea<br>Nut bar	Tea<br>Olives	Tea	Tea<br>Olives	Tea
**Dinner**	Minestrone Soup<br>Choco crisp bar<br>Vitamin capsule	Tea<br>Quinoa Mix Soup<br>Choco crisp bar<br>Vitamin capsule	Tea<br>Minestrone Soup<br>Vitamin capsule	Tea<br>Quinoa Mix Soup<br>Choco crisp bar<br>Vitamin capsule	Tea<br>Minestrone Soup<br>Vitamin capsule
**During the day**		Syrup for water flavouring	Syrup for water flavouring	Syrup for water flavouring	Syrup for water flavouring

Appendix 1. Example meal plan of the fasting-mimicking diet for study participants in the FIT trial

Appendix 2

CONSORT 2010 checklist

CONSORT 2010 checklist of information to include when reporting a randomised trial*

Section/Topic	Item No	Checklist item	Reported on page No
**Title and abstract**			
	1a	Identification as a randomised trial in the title	NA
	1b	Structured summary of trial design, methods, results, and conclusions (for specific guidance see CONSORT for abstracts)	p.2
**Introduction**			
Background and objectives	2a	Scientific background and explanation of rationale	p.3-5
	2b	Specific objectives or hypotheses	p.4-5
**Methods**			
Trial design	3a	Description of trial design (such as parallel, factorial) including allocation ratio	p.4 / ref 1
	3b	Important changes to methods after trial commencement (such as eligibility criteria), with reasons	NA
Participants	4a	Eligibility criteria for participants	p.5
	4b	Settings and locations where the data were collected	p.5

Interventions	5	The interventions for each group with sufficient details to allow replication, including how and when they were actually administered	p.5 / ref 1 / ref 2
Outcomes	6a	Completely defined pre-specified primary and secondary outcome measures, including how and when they were assessed	p.6-9
	6b	Any changes to trial outcomes after the trial commenced, with reasons	NA
Sample size	7a	How sample size was determined	NA (ref 1)
	7b	When applicable, explanation of any interim analyses and stopping guidelines	NA
Randomisation:			
Sequence generation	8a	Method used to generate the random allocation sequence	ref 1 / ref 2
	8b	Type of randomisation; details of any restriction (such as blocking and block size)	ref 1 / ref 2
Allocation concealment mechanism	9	Mechanism used to implement the random allocation sequence (such as sequentially numbered containers), describing any steps taken to conceal the sequence until interventions were assigned	ref 1 / ref 2
Implementation	10	Who generated the random allocation sequence, who enrolled participants, and who assigned participants to interventions	ref 1 / ref 2
Blinding	11a	If done, who was blinded after assignment to interventions (for example, participants, care providers, those assessing outcomes) and how	ref 1 / ref 2
	11b	If relevant, description of the similarity of interventions	NA
Statistical methods	12a	Statistical methods used to compare groups for primary and secondary outcomes	p.7

	12b	Methods for additional analyses, such as subgroup analyses and adjusted analyses	NA
**Results**			
Participant flow (a diagram is strongly recommended)	13a	For each group, the numbers of participants who were randomly assigned, received intended treatment, and were analysed for the primary outcome	Fig 1
	13b	For each group, losses and exclusions after randomisation, together with reasons	Fig 1 / ref 2
Recruitment	14a	Dates defining the periods of recruitment and follow-up	p.5 / ref 2
	14b	Why the trial ended or was stopped	NA
Baseline data	15	A table showing baseline demographic and clinical characteristics for each group	Table 1
Numbers analysed	16	For each group, number of participants (denominator) included in each analysis and whether the analysis was by original assigned groups	Table 2 / p.10
Outcomes and estimation	17a	For each primary and secondary outcome, results for each group, and the estimated effect size and its precision (such as 95% confidence interval)	Table 2
	17b	For binary outcomes, presentation of both absolute and relative effect sizes is recommended	NA
Ancillary analyses	18	Results of any other analyses performed, including subgroup analyses and adjusted analyses, distinguishing pre-specified from exploratory	Fig. 3
Harms	19	All important harms or unintended effects in each group (for specific guidance see CONSORT for harms)	NA / ref 2
**Discussion**			

Limitations	20	Trial limitations, addressing sources of potential bias, imprecision, and, if relevant, multiplicity of analyses	p.17
Generalisability	21	Generalisability (external validity, applicability) of the trial findings	p.17-18
Interpretation	22	Interpretation consistent with results, balancing benefits and harms, and considering other relevant evidence	p.15-18
**Other information**			
Registration	23	Registration number and name of trial registry	p.6
Protocol	24	Where the full trial protocol can be accessed, if available	ref 1
Funding	25	Sources of funding and other support (such as supply of drugs), role of funders	p.20

Citation: Schulz KF, Altman DG, Moher D, for the CONSORT Group. CONSORT 2010 Statement: updated guidelines for reporting parallel group randomised trials. BMC Medicine. 2010;8:18.

© 2010 Schulz et al. This is an Open Access article distributed under the terms of the Creative Commons Attribution License (http://creativecommons.org/licenses/by/2.0), which permits unrestricted use, distribution, and reproduction in any medium, provided the original work is properly cited.

*We strongly recommend reading this statement in conjunction with the CONSORT 2010 Explanation and Elaboration for important clarifications on all the items. If relevant, we also recommend reading CONSORT extensions for cluster randomised trials, non-inferiority and equivalence trials, non-pharmacological treatments, herbal interventions, and pragmatic trials. Additional extensions are forthcoming: for those and for up-to-date references relevant to this checklist, see www.consort-statement.org.

Reference 1: van den Burg EL, Schoonakker MP, van Peet PG, van den Akker-van Marle ME, Willems van Dijk K, Longo VD, et al. Fasting in diabetes treatment (FIT) trial: study protocol for a randomised, controlled, assessor-blinded intervention trial on the effects of intermittent use of a fasting-mimicking diet in patients with type 2 diabetes. BMC Endocr Disord. 2020;20(1):94.

Reference 2: van den Burg EL, Schoonakker MP, van Peet PG, van den Akker-van Marle ME, Lamb HJ, Longo VD, et al. Integration of a fasting-mimicking diet programme in primary care for type 2 diabetes reduces the need for medication and improves glycaemic control: a 12-month randomised controlled trial. Diabetologia. 2024. https://doi.org/10.1007/s00125-024-06137-0

Appendix 3

Semi-structured questionnaire for focus group discussions in the FIT trial

Fasting In diabetes Treatment
Experiences with following a fasting-mimicking diet: qualitative research

Introductory round

Analysed in the current study
During the study, you participated in the diet.
- How did you feel about the diet before the trial?
- What did you hope to achieve by following the diet?
- How many cycles of the diet did you complete?
- When did you complete the follow-up of the FIT study?

Theme 1: Compliance with the fasting-mimicking diet

Will be described elsewhere

General questions
- Can you tell me about your experience with the diet?
- Before starting the study, did you expect to complete the 12 dietary cycles?

Specific questions
1. Barriers and facilitators
- What made it difficult for you to adhere to the diet?
- What made it easier for you to adhere to the diet?
- Did the COVID-19 period affect your ability to maintain the diet?

2. Possible role of primary healthcare professionals
- What could help you to maintain the diet?
- Is there a potential role for primary healthcare professionals?

Possible topics to address if the discussion stalls
Possible factors that could play a role in whether or not the diet is maintained:
- Form of the diet (a ready-made box)
- Short duration (five days per month)
- Taste
- Side effects
- Fixed dates for the diet weeks
- Telephone appointments during the diet week
- Outpatient appointment on day 6 of the diet week
- Influence of weight change
- Influence of change in laboratory results (HbA1c, glucose)
- Impact of other health problems
- Environmental factors

- o Role of partner/spouse
- o Role of friends/family

-- Break --

Theme 2: Impact of following the fasting-mimicking diet on lifestyle.

Analysed in the current study

General questions

- Can you tell us something about your lifestyle during the year in which you participated in the FIT trial?
- Did following the diet affect your lifestyle?
- Were you motivated to change your lifestyle when you started the FIT trial?
- Did you plan to make other lifestyle changes during the study and did you feel you would succeed in doing so?
- What made it more difficult for you to change your lifestyle?
- What made it easier for you to change your lifestyle?

Specific questions

1. Influence on dietary pattern

- Did your usual eating pattern change during the time you regularly followed the diet?
- Did following the diet make you think differently about nutrition?
- Was there a period of "overeating" / binge eating / more snacking between diet periods?

2. Influence on exercise pattern

- Did your normal exercise pattern change during the time you regularly followed the diet?
- Did following the diet change your thoughts about exercise?
- Did you have enough energy to keep moving during the dietary cycles?

3. Possible role of healthcare professionals

- What might help to modify your usual lifestyle while following the diet intermittently?
- Is there possibly a role for primary care professionals?

4. Lifestyle during the corona period

- Did the COVID-19 period affect your lifestyle?

-- End --

Appendix 4

Analyses of the sub-scores of the Eetscore FFQ over time

	FMD group		Control group		Estimated effect (95% CI)	p-value
	n	Mean (SD)	n	Mean (SD)		
**Vegetables (DHD1)**						
Baseline	47	7.2 (2.8)	43	6.2 (3.3)		
6 months	45	6.9 (3.0)	39	6.0 (3.0)	-0.3 (-1.3 to 0.8)	0.65
12 months	45	7.0 (2.7)	40	5.8 (3.5)	0.1 (-1.0 to 1.2)	0.84
**Fruit (DHD2)**						
Baseline	47	6.2 (2.9)	43	5.5 (3.6)		
6 months	45	6.0 (3.2)	39	5.6 (3.6)	-0.4 (-1.4 to 0.7)	0.50
12 months	45	6.4 (3.2)	40	5.7 (3.4)	-0.1 (-1.1 to 1.0)	0.92
**Wholegrain products (DHD3)**						
Baseline	47	7.6 (2.8)	43	7.0 (2.9)		
6 months	45	7.3 (3.0)	39	7.2 (3.2)	-0.4 (-1.6 to 0.7)	0.46
12 months	45	6.9 (2.6)	40	6.8 (2.8)	-0.4 (-1.6 to 0.7)	0.45
**Legumes (DHD4)**						
Baseline	47	5.9 (4.5)	43	6.0 (4.5)		
6 months	45	4.8 (4.6)	39	5.8 (4.5)	-0.9 (-2.7 to 0.8)	0.29
12 months	45	5.7 (4.4)	40	6.1 (4.2)	-0.3 (-2.0 to 1.4)	0.72
**Nuts (DHD5)**						
Baseline	47	4.8 (3.7)	43	5.9 (3.7)		
6 months	45	5.2 (3.8)	39	5.7 (4.0)	0.6 (-0.7 to 1.9)	0.35
12 months	45	5.2 (3.5)	40	6.0 (3.7)	0.1 (-1.2 to 1.4)	0.92
**Dairy (DHD6)**						
Baseline	47	6.7 (3.1)	43	5.5 (3.6)		
6 months	45	6.5 (3.4)	39	5.5 (3.7)	-0.3 (-1.5 to 1.0)	0.68
12 months	45	5.7 (3.2)	40	4.7 (3.6)	0.1 (-1.2 to 1.3)	0.90
**Fish (DHD7)**						
Baseline	47	6.1 (3.6)	43	7.5 (3.0)		
6 months	45	6.3 (3.4)	39	7.0 (3.4)	0.8 (-0.3 to 1.9)	0.17
12 months	45	7.1 (3.2)	40	7.5 (3.3)	0.9 (-0.2 to 2.0)	0.12
**Tea (DHD8)**						
Baseline	47	4.6 (4.4)	43	2.1 (3.1)		
6 months	45	4.2 (4.6)	39	2.7 (3.7)	-1.2 (-2.5 to 0.1)	0.08
12 months	45	3.9 (4.2)	40	2.5 (3.9)	-1.2 (-2.5 to 0.1)	0.08
**Fats and oils (DHD9)**						
Baseline	47	5.3 (4.7)	43	5.3 (4.7)		
6 months	45	4.8 (4.7)	39	5.7 (4.7)	-0.8 (-2.7 to 1.2)	0.46
12 months	45	5.6 (4.8)	40	4.6 (4.8)	1.2 (-0.8 to 3.2)	0.23
**Coffee (DHD10)**						
Baseline	47	7.1 (2.5)	43	7.4 (2.5)		
6 months	45	7.6 (2.5)	39	7.8 (2.5)	0.2 (-0.7 to 1.0)	0.72
12 months	45	7.6 (2.5)	40	7.6 (2.5)	0.3 (-0.5 to 1.1)	0.49
**Red meat (DHD11)**						
Baseline	47	8.5 (2.8)	43	8.1 (3.2)		
6 months	45	8.9 (2.6)	39	8.0 (3.6)	0.5 (-0.7 to 1.6)	0.45
12 months	45	8.8 (2.7)	40	8.1 (3.7)	0.3 (-0.9 to 1.5)	0.63
**Processed meat (DHD12)**						
Baseline	47	3.2 (3.2)	43	4.0 (3.7)		
6 months	45	4.3 (3.0)	39	4.8 (3.8)	0.3 (-0.8 to 1.4)	0.60
12 months	45	3.4 (3.3)	40	4.9 (3.7)	-0.8 (-1.9 to 0.3)	0.15
**Sweetened beverages and fruit juices (DHD13)**						
Baseline	47	8.1 (3.5)	43	8.0 (3.2)		
6 months	45	8.1 (3.2)	39	8.4 (2.9)	-0.2 (-1.5 to 1.1)	0.78

12 months	45	8.6 (2.8)	40	8.8 (2.4)	-0.2 (-1.5 to 1.2)	0.81
**Alcohol (DHD14)**						
Baseline	47	8.7 (2.8)	43	7.8 (3.6)		
6 months	45	8.7 (2.6)	39	8.0 (3.1)	-0.2 (-1.1 to 0.7)	0.61
12 months	45	8.8 (2.4)	40	8.4 (2.9)	-0.5 (-1.4 to 0.4)	0.27
**Salt (DHD15)**						
Baseline	47	8.3 (2.3)	43	7.2 (3.2)		
6 months	45	8.5 (2.0)	39	7.5 (3.0)	-0.1 (-1.0 to 0.8)	0.81
12 months	45	8.5 (2.0)	40	8.1 (2.3)	-0.7 (-1.6 to 0.2)	0.14
**Unhealthy choices (DHD16)**						
Baseline	47	6.1 (4.1)	43	5.3 (4.4)		
6 months	45	5.0 (4.4)	39	5.4 (4.3)	-0.9 (-2.4 to 0.6)	0.26
12 months	45	5.3 (4.3)	40	4.9 (4.4)	-0.1 (-1.6 to 1.5)	0.94

Appendix 4. Analyses of the sub-scores of the Eetscore FFQ over time using linear mixed models (intention-to-treat analysis).

For each food component a score is calculated ranging between 0 to 10, indicating minimal to maximum adherence to the advices from the Dutch dietary guidelines of 2015, as defined by the Dutch Health Council of the Netherlands.

Linear mixed models were computed with time, intervention and time*intervention interaction as fixed-effects, and individual participants as random effect.

CI = confidence interval. DHD = food component of the Dutch Healthy Diet 2015-index. FMD = fasting-mimicking diet. SD = standard deviation.

Appendix 5

Characteristics of focus group participants compared to the other FMD participants

	Participants focus groups (*n* = 20)	Other FMD participants (*n* = 29)	p-value
**Demographics**			
Age (years), mean ± SD	63.0 ± 8.2	63.9 ± 8.1	0.63
Sex, *n* (%)			0.78
Male	10 (50.0)	16 (55.2)	
Female	10 (50.0)	13 (44.8)	
Level of education, *n* (%)			0.67
Low	7 (35.0)	13 (44.8)	
Intermediate	5 (25.0)	8 (27.6)	
High	7 (35.0)	7 (24.1)	
Unknown	1 (5.0)	1 (3.4)	
**Type 2 diabetes**			
Time since diagnosis T2D (years), median (IQR)	4.5 (2.0 – 10.3)	4.0 (3.0 – 12.0)	0.85
HbA1c (mmol/mol), mean ± SD	50.8 ± 8.1	53.1 (10.0)	0.46
Use of glucose-lowering medication			
Metformin, *n* (%)	18 (90.0)	28 (96.6)	0.56
**Anthropometrics**			
BMI (kg/m^2), median (IQR)	30.9 (28.6 – 33.1)	33.1 (29.6 – 38.3)	0.12
**FIT trial**			
Number of completed FMD cycles, median (IQR)	12 (12 – 12)	11 (4 – 12)	0.02

Appendix 5. Characteristics of participants in the focus groups (n=20) compared to the other FMD participants who did not join the focus groups (n=29).
Data are presented as mean ± SD, median (IQR) or number (*n*) with percentage (%).

BMI = Body Mass Index. FMD = fasting-mimicking diet. HbA1c = glycated haemoglobin. IQR = interquartile range. n = number. SD = standard deviation. T2D = type 2 diabetes.

Appendix 6

Results from focus group discussions

All barriers and facilitators involved in additional self-initiated lifestyle changes while following an FMD, identified during six focus group discussions. Barriers and facilitators are mapped onto the combined Capability, Opportunity, and Motivational Behaviour (COM-B) model and the Theoretical Domain Framework (TDF). A '+' indicates a facilitator, a '-' indicates a barrier.

**COM-B Model**<br>*Corresponding TDF domain*	**Barriers and facilitators for lifestyle change**
**Capability**	
Physical	FMD related factors:<br>+ Feeling less hunger or feeling satiated much faster influences eating behaviour<br>+ Improvement in physical fitness stimulating more physical activity
	FMD unrelated factors:<br>- Existing health problems limit physical activity
Knowledge	FMD related factors:<br>+ Improving knowledge, such as by reading information on aspects of the FMD<br>+ Increased awareness of the importance of a healthy lifestyle (including dietary changes and increasing physical activity), related to health improvements and type 2 diabetes
	FMD unrelated factors:<br>+ Improving knowledge, such as by watching food programs on television or information from other media<br>+ Other health problems (for example cardiac problems) stimulating awareness of the importance of a healthy lifestyle<br>- Disinformation from the past hindering emergence of new dietary patterns
Behavioural regulation	FMD related factors:<br>+ Experiencing different eating patterns, for example fewer eating moments during the day or reducing alcoholic beverages<br>+ Increased internal motivation to improve dietary and physical activity patterns
	FMD unrelated factors:<br>+ An existing healthy lifestyle ('healthy habits') makes it easier to continue a healthy lifestyle<br>- Existing unhealthy dietary patterns making it more difficult to start with new healthy eating patterns
**Opportunity**	
Environmental context and resources	FMD related factors:<br>+ Structure of FMD facilitating new healthy dietary patterns

	- Costs of the FMD making it difficult for participants to continue the FMD after the FIT trial
	FMD unrelated factors:<br>+ Context and resources encouraging (more) physical activity (examples are not having a car, having a dog or being retired)<br>+ Support from healthcare professionals promoting physical activity and healthier dietary patterns<br>+ Pleasant weather encouraging physical activity<br>+ Nature close by encouraging physical activity<br>- Restriction of physical activity due to COVID-19 virus outbreak, such as staying indoors more out of fear and working from home<br>- A sedentary or international work occupation hindering change in physical activity<br>- Rainy, cold and bad weather hindering physical activity
Social influences	FMD unrelated factors:<br>+ Family support and group exercise increasing physical activity<br>+ Family support encouraging changing dietary customs<br>- Social events that encouraging unhealthy eating<br>- Usual dietary patterns in family life, for example caused by teenagers in the house who have other needs than the participant<br>- Social factors, such as fear of failure in a walking group, limiting changing physical activity patterns
**Motivation**	
Emotion	FMD related factors:<br>+ Feeling fitter since following a FMD<br>+ Feeling satisfied with the results of following a FMD<br>- Felling dissatisfied with the results of following a FMD
	FMD unrelated factors:<br>- COVID-19 outbreak, causing mood problems which people gave as reason for increasing consumption of snacks and comfort food<br>- Temporary health problems, such as a shoulder fracture, causing mood problems which people gave as reason for increasing consumption of snacks and comfort food<br>- Negative events regarding family/friends (for example health problems) causing mood problems, which people gave as reason for increasing consumption of snacks and comfort food
Reinforcement	FMD related factors:<br>+ FMD-related weight loss, resulting in an increased capability to perform physical activity<br>+ FMD-related health improvements (examples are less medication and fewer physical symptoms)
	FMD unrelated factors:<br>+ Other health improvements facilitating a healthy lifestyle, for example recovering from anaemia
Intentions	FMD related factors:

	+ Intention to change dietary patterns<br>+ Intention to increase physical activity
	FMD unrelated factors:<br>+ Internal motivation, perseverance<br>- Lack of internal motivation
Goals	FMD related factors:<br>+ Having goals for the FMD period, for example to use less medication and achieve other health improvements, stimulating other lifestyle changes as well
	FMD unrelated factors:<br>+ Weight loss achieved by an increase in physical activity stimulating other lifestyle changes<br>+ Internal motivation, perseverance
